# Common mental disorders in Primary Health Care professionals during the COVID-19 pandemic period: a cross-sectional study in the Northern health macro-region of Minas Gerais state, Brazil, 2021

**DOI:** 10.1590/S2237-96222023000100012

**Published:** 2023-06-05

**Authors:** Fabrício Emanuel Soares de Oliveira, Samuel Trezena, Verônica Oliveira Dias, Hercílio Martelli, Daniella Reis Barbosa Martelli

**Affiliations:** 1 Universidade Estadual de Montes Claros, Programa de Pós-Graduação em Cuidado Primário em Saúde, Montes Claros, MG, Brazil

**Keywords:** Mental Disorders, Health Personnel, Primary Health Care, Covid-19, Cross-sectional studies., Trastornos Mentales, Personal de Salud, Atención Primaria de Salud, COVID-19, Estudios Transversales., Transtornos Mentais, Pessoal de Saúde, Atenção Primária à Saúde, Covid-19, Estudos Transversais.

## Abstract

**Objective:**

to analyze the prevalence of symptoms of common mental disorders (CMDs) in Primary Health Care professionals between August-October/2021.

**Methods:**

this was a cross-sectional study conducted with health professionals in the Northern health macro-region of Minas Gerais state; snowball sampling was used; the dependent variable, CMDs, was evaluated using the Self-Reporting Questionnaire (SRQ-20); Poisson regression was used to perform the statistical analysis.

**Results:**

a total of 702 health professionals took part in the study; the prevalence of CDMs was 43.2%. It was higher in those with previous [prevalence ratios (PR) = 2.42; 95%CI 1.43;4.08] and current (PR = 1.54; 95%CI 1.25;1.89) symptoms of mental disorders, overwork during the pandemic (PR = 1.42; 95%CI 1.16;1.73), previous symptoms of anxiety (PR = 1.27; 95%CI 1.01;1.61), depression (PR = 1.27; 95%CI 1.06;1.52) and other mental disorders (PR = 1.20; 95%CI 1.01;1.43).

**Conclusion:**

there was an association between CDMs and presenting previous and current symptoms of mental disorders and work overload during the covid-19 pandemic.

## Resumo

**Objetivo:**

analisar a prevalência de sintomas de transtornos mentais comuns (TMCs) em profissionais de saúde da Atenção Primária à Saúde, no período agosto-outubro/2021.

**Métodos:**

estudo transversal com profissionais de saúde da macrorregião Norte de saúde de Minas Gerais; amostragem de tipo “bola de neve”; a variável dependente, TMCs, foi avaliada pelo Self-Reporting Questionnaire (SRQ-20); utilizou-se regressão de Poisson na análise estatística.

**Resultados:**

participaram 702 profissionais de saúde; a prevalência de TMCs foi de 43,2%, maior naqueles que apresentaram sintomas de transtornos mentais prévios [razão de prevalências (RP) = 2,42 ;IC_95%_ 1,43;4,08] e atuais (RP = 1,54; IC_95%_ 1,25;1,89), trabalho a mais durante a pandemia (RP = 1,42; IC_95%_ 1,16;1,73), sintomas prévios de ansiedade (RP = 1,27; IC_95%_ 1,01;1,61), depressão (RP = 1,27; IC_95%_ 1,06;1,52) e outros transtornos mentais (RP = 1,20; IC_95%_ 1,01;1,43).

**Conclusão:**

observou-se associação de TMCs com sintomas prévios e atuais de transtornos mentais e sobrecarga de trabalho, durante a pandemia da covid-19.

## Resumen

**Objetivo:**

analizar la prevalencia de síntomas de trastornos mentales comunes (TMC) en profesionales sanitarios de la Atención Primaria de Salud de agosto a octubre de 2021.

**Métodos:**

estudio transversal realizado con profesionales de la salud de la macrorregión norte de Minas Gerais. El muestreo fue del tipo bola de nieve. La variable dependiente, TMC, se evaluó mediante el *Self-Reporting Questionnaire* (SRQ-20). En el análisis estadístico se utilizó la regresión de Poisson.

**Resultados:**

participaron 702 profesionales de salud. La prevalencia de TMC fue del 43,2%, mayor en quienes presentaban síntomas de trastornos mentales previos (RP = 2,42; IC_95%_ 1,43;4,08) y actuales (RP = 1,54; IC_95%_ 1,25;1,89); exceso de trabajo durante la pandemia (RP = 1,42; IC_95%_ 1,16;1,73); síntomas previos de ansiedad (RP = 1,27; IC_95%_ 1,01;1,61), depresión (RP = 1,27; IC_95%_ 1,06;1,52) y otros trastornos mentales (RP = 1,20; IC_95%_ 1,01;1,43).

**Conclusión:**

hubo una asociación entre los TMC y los síntomas previos y actuales de los trastornos mentales y la sobrecarga de trabajo durante la pandemia de COVID-19.

**Table:** 

Study contributions	
**Main results**	Among 702 health professionals, the prevalence of common mental disorders (CMDs) was 43.2%; there was a higher prevalence in females, frontline workers, those who worked more than usual, and those with previous and current symptoms of mental disorders.
**Implications for services**	The prevalence of CMDs and the associated factors identified indicate that healthcare workers need care and improvement in their working conditions.
**Perspectives**	The need for mental health support for health professionals was observed. Interventions aimed at mental health, related to new work processes and follow-up of individuals with symptoms of mental disorders, are suggested.

## INTRODUCTION

Common mental disorders (CMDs) are characterized by non-psychotic symptoms, including depressive symptoms, anxiety symptoms, irritability, insomnia, forgetfulness, difficulty concentrating and somatic symptoms (physical symptoms with psychological causes), and can cause impairment in functional capacity, although they are often not classified according to a diagnosis specified in a nosological manual.^1^

The worldwide prevalence of CMDs in the general population was estimated at 29.2% [95% confidence interval (95%CI) 25.9;32.6], according to a systematic review that evaluated studies between 1980 and 2013.^2^ In Brazil, another systematic review published in 2022,^3^ found similar results, in which the prevalence of CMDs was 30.0% (95%CI 27.0;34.0).

Mental illness has had an increasing impact on the health of humanity. In 2013, the Global Burden of Disease estimated that worldwide, of the total years lived with disability, 21.2% were caused by mental disorders.^4^ In 2016, this estimate increased to 32.4%,^4^ characterized not only as a public health problem, but also as a reflection of social and economic aspects.^4^

In Brazil, on February 26, 2020, the first case of COVID-19 was confirmed, and on March 11, 2020, the World Health Organization (WHO) declared the disease a pandemic.^5^ A study that evaluated the response capacity of health systems in 182 countries in the context of the pandemic, observed that only half of them had strong operational readiness capacities in place to cope with emergency situations. As COVID-19 progressed and resources became scarce, health services had to reorganize their work process in order to meet the new demands imposed by the pandemic, exposing health professionals to stress factors.^6^

At the Primary Health Care (PHC) level within the Brazilian National Health System (*Sistema Único de Saúde -* SUS), several routine activities were suspended due to the COVID-19 pandemic, with priority care being maintained, such as vaccination, the monitoring of people with chronic and priority diseases (e.g.: pregnant women) and acute cases.^7^ PHC played a fundamental role in increasing the response capacity to the dissemination of COVID-19, because at this level of care, health surveillance actions, vaccination campaigns, case monitoring and referral to other levels of care are performed.^7^

Studies show that mental health problems are common among health professionals.^8^ ^),(^ ^9^ ^)^ During the pandemic, there were significant changes in the routine of these professionals, such as increased workload, social isolation and fear of infection and transmission, factors that may be associated with the symptoms of CMDs.^10^

This study aimed to analyze the prevalence of symptoms of CMDs and associated factors in PHC professionals in a given period of the COVID-19 pandemic, that is, between August and October 2021, in the Northern health macro-region of the state of Minas Gerais, Brazil.

## METHODS

This was an observational, cross-sectional and quantitative study. The research was conducted with professionals in the Northern health macro-region of Minas Gerais state, which is comprised of 54 municipalities in the catchment area of the Regional Health Superintendence (*Superintendência Regional de Saúde* - SRS) of Montes Claros, 25 municipalities in the catchment area of the Regional Health Management (*Gerência Regional de Saúde* - GRS) of Januária and 7 municipalities in the catchment area of the GRS of Pirapora, totaling 86 municipalities.^11^

The Northern health macro-region of Minas Gerais state, with a territorial extension of 103,660.5km^2^, had 1,676,413 inhabitants according to the 2020 Minas Gerais Health Regionalization Master Plan. The 86 municipalities that make up this region are organized into 11 microregions.^11^

The research participants were PHC professionals in the Northern health macro-region of Minas Gerais state, selected according to the following inclusion criterion: being a PHC professional who works in the cities that make up the Northern health macro-region of Minas Gerais state. The exclusion criterion was to be on vacation or away from work, for any reason, during the data collection period.

The sample size calculation was performed using the OpenEpi platform.^12^ On August 1, 2021, the TABNET system, developed by the Brazilian National Health System Information Technology Department (*Departamento de Informática do Sistema Único de Saúde* - DATASUS), found a total of 8,968 health professionals registered in the Northern health macro-region of Minas Gerais state. We adopted the statistical parameters of estimated prevalence of 32%,^8^ 95% confidence level, margin of error of 5% and correction by the design effect (deff = 2), obtaining a minimum sample of 645 health professionals.

In this study, we opted for a “snowball” sampling technique, using an online questionnaire, given the social isolation imposed by the covid-19 pandemic. Snowball sampling is a type of sampling widely used in qualitative studies; however, it has recently been used in quantitative and virtual studies, especially during the pandemic.^13^ ^)-(^ ^15^

The SRS in Montes Claros and the GRS in Januária and Pirapora provided the telephone numbers and e-mails of the PHC coordinators of their respective municipalities on July 15, 2021; subsequently, we made contact with the PHC coordinators from the 86 municipalities in the region and, invitations to take part in the study were sent via e-mail and social networks (WhatsApp^®^ and Instagram^®^). The coordinators forwarded the invitations - with the link to access the questionnaire - to health professionals in their respective municipalities; these invitations also requested that the e-mail be sent to other PHC professionals. Three attempts were made to contact the PHC coordinators in each municipality.

Data collection occurred between August and October 2021, by completing an online questionnaire made available to participants using the Google Forms^®^. The questionnaire was comprised of the following independent variables and response options:

a) sex (male; female);
b) age (open-ended response, subsequently categorized as less than or equal to 34 years old greater than or equal to 35 years old, according to the distribution of the results);
c) marital status (single, married, widowed, divorced, subsequently classified as with a partner or without a partner);
d) profession [community health agent (CHA); social worker; dentist; physical educator; nurse; pharmacist; physiotherapist; speech therapist; physician; nutritionist; psychologist; nursing technician; others (open response field), subsequently categorized as middle /technical level or higher level];
e) municipality where he or she works (open-ended response, subsequently categorized according to GRS/SRS);
f) working on the front line in the fight against COVID-19 (yes; no);
g) contagion by covid-19 (yes; no);
h) previous symptoms of CMDs at some point in life (yes; no);
i) current symptoms of CMDs (yes; no);
j) type of disorder presented at some point in life (open-ended response, subsequently categorized as anxiety symptoms: yes; no);
k) depressive symptoms (yes; no);
l) insomnia symptoms (yes; no);
m) other symptoms of mental disorders (yes; no);
n) previous psychological or psychiatric follow-up at some point in life (yes; no);
o) current psychological or psychiatric follow-up (yes; no);
p) previous use of psychotropic medication at some point in life (yes; no);
q) current use of psychotropic medication (yes; no);
r) type of medication used at some point in life (open-ended response, subsequently categorized as: antidepressants; anxiolytics; another type of psychotropic medication); and
s) work during the pandemic (choice options for the participating professional: “*In the same way or less than usual”; “ More than usual and I felt overwhelmed*”).

CMDs were assessed using the Self-Reporting Questionnaire (SRQ-20). This is an instrument developed by the WHO, which was designed to screen for CMDs. Consisting of 20 questions that have to be answered by “yes” or “no”, the SRQ-20 evaluates four groups of symptoms: depressive-anxious mood; somatic symptoms; decrease in vital energy; and depressive thoughts. In order to define the variable, we used a cutoff point of 7, which presents sensitivity and specificity of 86.3% and 89.3%, respectively.^16^ Thus, participants who presented a score equal to or greater than 7 were classified with CMD. After answering the questionnaire, the participants had access to a booklet developed by the research team, with information on basic mental health care, directed to health professionals. The booklet can be found at: https://drive.google.com/file/d/1hQtTCzNyVMFOI7g9sWiaVsTWlp2cB9Bu/view

A database was built using the Statistical Package for the Social Sciences, for Windows, Inc., USA (SPSS^®^) version 24.0, to perform statistical analyses. As non-probability sampling makes it impossible to know the natural weights of the sampling design, a post-stratification weighting procedure was applied in order to improve the representativeness of the sample. The reference population of this study (total of PHC health professionals in the Northern health macro-region of Minas Gerais state) was stratified in the professional categories with the highest number of workers (nurse; physiotherapist; doctor; dentist; psychologist; nursing technician; community health agent; other categories grouped into a single stratum for having few professionals), according to information available in the information system of the Brazilian National Health System (TABNET), and the same procedure was performed with the study sample. In order to calculate the weighting factor (weight), we used the following formula:

P = (Ne/ne)x (n/N)

Where:

W = weight

Ne = number of professionals in each professional category in the population

ne = number of professionals in each professional category in the sample

n = sample size

N = total population size

The weighting method used was based on the study by Szwarcwald et al.^17^ After calculating the weights, the weight variable was created in SPSS, performing the analysis using weight cases function by the weight variable.

With the “weight cases” function activated, we performed a descriptive analysis with the frequency of all variables, mean and standard deviation of the variable “age”, followed by bivariate analysis by means of simple Poisson regression to calculate the prevalence ratio (PR) with confidence intervals. Finally, multiple Poisson regression model with robust variance was performed through the commands for analyzing generalized linear models, using weights as the scale weighting variable, in which the variables that presented significance level of up to 0.20 in the bivariate analysis were included, remaining in the final model those that had an association at 5% level (p-value ≤ 0.05). The quality of model adjustment was assessed using the deviance test; and multilinearity, by means of variance inflation factor (VIF) and tolerance.

The study followed the guidelines of the National Health Council (Conselho Nacional de Saúde - CNS) of the Ministry of Health, Resolution No. 466, of December 12, 2012. The Free and Informed Consent Form (FICF), the invitation to participate and the way to contact the participants followed the guidelines of Circular Letter No. 1/2021 of the National Research Ethics Committee (*Comissão Nacional de Ética em Pesquisa* - CONEP), which provides guidelines for research in virtual environments. The study project was approved by the Research Ethics Committee of the Universidade Estadual de Montes Claros (CEP/Unimontes) on July 9, 2021: Opinion No. 4,838,846. Certificate of Submission for Ethical Appraisal (*Certificado de Apresentação para Apreciação Ética* - CAAE) No. 47795821.7.0000.5146. All participants signed the FICF before having access to the questionnaire.

## RESULTS

The data collection stage resulted in a final sample of 702 health professionals, working in 61 of the 86 municipalities of the Northern health macro-region of Minas Gerais state. There were no answers after three attempts to contact professionals from 25 municipalities.

Regarding the sociodemographic variables, it could be seen that the majority of the participants were female (84.6%), 50.4% were aged up to 34 years, the average age was 35.3 years and more than half of them (55.3%) were married. As for the characteristics related to professional performance, more than half of the professionals reported working in the SRS of Montes Claros (63.3%), as well as in a middle or technical level job (59.3%) (Table 1).

**Table 1:** Characterization of participants and results of the bivariate analysis among common mental disorders according to the Self-Reporting Questionnaire and study variables, in health professionals (n = 702) in the Northern health macro-region of Minas Gerais state, Brazil, 2021

Variables	Total	Absence of CMDs^b^	Presence of CMDs^b^	PR^c^ (95%CI)^a^	p-value
	% (95%CI)^a^	% (95%CI)^a^	% (95%CI)^a^		
Sex					
Male	15.4 (12.9;18.2)	72.2 (63.1;79.8)	27.8 (20.2;36.9)	1.00	
Female	84.6 (81.7;87.1)	54.0 (50.0;58.0)	46.0 (41.2;50.0)	1.63 (1.13;2.36)	0.008
Age (in full years)					
≥ 35	49.6 (45.9;53.2)	58.0 (52.8;63.1)	42.0 (36.9;47.2)	1.00	
≤ 34	50.4 (46.7;54.1)	55.4 (50.1;60.4)	44.6 (39.5;49.8)	1.06 (0.87;1.28)	0.533
Marital status					
Single/widowed/divorced	44.7 (41.0;48.4)	56.4 (50.8;61.8)	43.6 (38.2;49.1)	1.03 (0.85;1.25)	
Married	55.3 (51.6;58.9)	57.8 (52.8;62.6)	42.2 (37.4;47.2)	1.00	0.729
Performance level					
Middle/technical level	59.3 (55.5;62.8)	60.6 (55.8;65.1)	39.4 (34.8;44.2)	1.00	
Higher level	40.7 (37.1;44.4)	51.4 (45.6;57.1)	48.6 (42.8;54.3)	1.23 (1.01;1.48)	0.031
Region					
Januária GRS^d^/Pirapora GRS^d^	36.7 (33.2;40.3)	61.3 (55.2;67.1)	38.7 (32.9;44.7)	1.00	
Montes Claros SRS^e^	63.3 (59.6;66.7)	54.2 (49.5;58.8)	45.8 (41.2;50.4)	1.18 (0.95;1.46)	0.118
Overall prevalence	-	56.8 (53.1;60.4)	43.2 (39.5;46.8)	-	-

a) 95%CI: 95% confidence interval; b) CMDs: Common mental disorders; c) PR: Prevalence ratio; d) GRS (Gerência Regional de Saúde): Regional Health Management; e) SRS (Superintendência Regional de Saúde): Regional Health Superintendence.

Most of them reported working on the front line in the fight against COVID-19 (74.4%) and having worked more during the pandemic (51.8%). In addition, almost a quarter of participants (22.9%) reported having already been diagnosed with COVID-19. Presence of symptoms of previous mental disorders was reported by 67.3% of the sample; anxiety symptoms were the most frequently reported symptoms (55.9%). About one third of the participants reported having received psychological or psychiatric follow-up at some point in their lives. Current use of psychotropic medication was less frequently reported than previous use of these medication (22.4% versus 37.3%, respectively). Regarding the use of psychotropic medications, antidepressants were the most commonly used (21.7%) (Table 2).

**Table 2:** Characterization of participants regarding covid-19, work, mental health condition and result of the bivariate analysis among common mental disorders according to the Self-Reporting Questionnaire, in health professionals (n = 702) in the Northern health macroregion of Minas Gerais state, Brazil, 2021

Variables	Total	Absence of CMDs^b^	Presence of CMDs^b^	PR^c^ (95%CI)^a^	p-value
	% (95%CI)^a^	% (95%CI)^a^	% (95%CI)^a^		
Working on the front line in the fight against covid-19					
No	25.6 (22.5;29.0)	64.6 (57.2;71.2)	35.4 (28.7;42.7)	1.00	
Yes	74.4 (70.9;77.5)	53.3 (49.0;57.6)	46.7 (42.3;51.0)	1.31 (1.01;1.69)	0.035
Previous diagnosis of covid-19					
No	77.1 (73.8;80.0)	59.3 (55.1;63.4)	40.7 (36.6;44.8)	1.00	
Yes	22.9 (19.9;26.2)	48.4 (40.8;56.1)	51.6 (43.9;59.1)	1.27 (1.03;1.55)	0.019
Presence of previous mental disorder symptoms					
No	32.7 (29.2;36.2)	88.9 (84.1;92.4)	11.1 (7.6;15.8)	1.00	
Yes	67.3 (63.7;70.7)	40.8 (36.4;45.3)	59.2 (54.7;63.6)	5.33 (3.48;8.14)	< 0.001
Presence of current mental disorder symptoms					
No	64.4 (60.7;67.8)	74.3 (70.0;78.1)	25.7 (21.8;30.0)	1.00	
Yes	35.6 (32.1;39.2)	25.7 (20.6;31.5)	74.3 (68.4;79.3)	2.88 (2.36;3.52)	< 0.001
Previous psychological or psychiatric follow-up					
No	64.3 (60.6;67.7)	64.3 (59.7;68.5)	35.7 (31.4;40.2)	1.00	
Yes	35.7 (32.2;39.3)	43.0 (36.7;49.1)	57.0 (50.8;63.0)	1.59 (1.32;1.91)	< 0.001
Current psychological or psychiatric follow-up					
No	86.6 (83.9;88.9)	59.8 (55.8;63.6)	40.2 (36.3;44.1)		
Yes	13.4 (11.0;16.1)	36.6 (27.5;46.7)	63.4 (53.3;72.5)	1.57 (1.28;1.93)	< 0.001
Previous psychotropic medication use					
No	62.7 (59.0;66.1)	69.6 (65.1;73.7)	30.4 (26.2;34.8)	1.00	
Yes	37.3 (33.8;40.9)	34.9 (29.3;40.8)	65.1 (59.1;70.6)	2.14 (1.77;2.59)	< 0.001
Current psychotropic medication use					
No	77.6 (74.3;80.5)	64.3 (60.1;68.3)	35.7 (31.7;39.8)	1.00	
Yes	22.4 (19.4;25.6)	30.7 (23.9;38.4)	69.3 (61.5;76.0)	1.94 (1.62;2.32)	< 0.001
Work during the pandemic					
I worked the same way or less than usual	48.2 (44.4;51.9)	67.9 (62.6;72.7)	32.1 (27.2;37.4)	1.00	
I worked more than usual and I felt overwhelmed	51.8 (48.0;55.5)	43.8 (38.6;49.0)	56.2 (50.9;61.3)	1.75 (1.42;2.16)	< 0.001
Previous self-reported anxiety symptoms					
No	44.1 (40.4;47.8)	79.7 (74.8;83.8)	20.3 (16.2;25.2)	1.00	
Yes	55.9 (52.1;59.5)	38.3 (33.6;43.3)	61.7 (56.7;66.3)	3.02 (2.33;3.92)	< 0.001
Previous self-reported insomnia symptoms					
No	77.3 (74.0;80.2)	62.7 (58.5;66.7)	37.3 (33.2;41.4)	1.00	
Yes	22.7 (19.7;26.0)	35.7 (28.6;43.4)	64.3 (56.5;71.4)	1.72 (1.44;2.06)	< 0.001
Previous self-reported depressive symptoms					
No	87.8 (85.2;90.0)	61.6 (57.7;65.4)	38.4 (34.6;42.3)	1.00	
Yes	12.2 (9.9;14.8)	20.2 (13.0;30.0)	79.8 (69.9;86.7)	2.07 (1.74;2.45)	< 0.001
Other previous self-reported symptoms					
No	90.4 (88.0;92.4)	60.6 (56.7;64.4)	39.4 (35.6;43.2)	1.00	
Yes	9.6 (7.5;11.9)	18.2 (10.7;29.1)	81.8 (70.8;89.2)	2.09 (1.78;2.45)	< 0.001
Previous self-reported antidepressant medication use					
No	78.3 (75.1;81.2)	63.3 (59.1;67.2)	36.7 (32.8;40.8)	1.00	
Yes	21.7 (18.7;24.8)	33.6 (26.5;41.3)	66.4 (58.6;73.4)	1.80 (1.51;2.16)	< 0.001
Previous self-reported anxiolytic medication use					
No	79.2 (76.0;82.0)	63.4 (59.3;67.3)	36.6 (32.6;40.6)	1.00	
Yes	20.8 (17.9;24.0)	31.5 (24.5;39.4)	68.5 (60.5;75.4)	1.87 (1.56;2.23)	< 0.001
Previous self-reported use of another psychotropic medication					
No	92.0 (88.7;93.8)	59.1 (55.2;62.8)	40.9 (37.2;44.7)	1.00	
Yes	8.0 (6.2;10.2)	30.4 (19.9;43.3)	69.6 (56.6;80.1)	1.69 (1.35;2.12)	< 0.001

a) 95%CI: 95% confidence interval; b) CMDs: Common mental disorders; c) PR: Prevalence ratio.

The overall prevalence of CMDs was 43.2%. Tables 1 and 2 also show the prevalence of CMDs and PR by variable. The following variables were considered for the multiple model (p-value ≤ 0.20): sex; income; performance level; region of residence; working on the frontline in the fight against COVID-19; previous diagnosis of COVID-19; presence of previous and current mental disorder symptoms; previous and current psychological/psychiatric follow-up; previous and current psychotropic medication use; worked during the pandemic; previous self-reported anxiety symptoms; previous self-reported insomnia symptoms; previous self-reported depressive symptoms; other previous self-reported symptoms; previous self-reported antidepressant medication use; previous self-reported anxiolytic medication use; and previous self-reported use of another psychotropic medication. These variables were included in the final model of multiple analysis.

Regarding the professions of the participants, 18 professional categories of middle, technical and higher levels were identified, with a greater participation of CHAs (31.6%), followed by nurses (23.9%), dentists (13.0%) and nursing technicians (11.0%) (Table 3).

**Table 3:** Characterization of participants (n = 702) by profession, Northern health macro-region of Minas Gerais state, Brazil, 2021

Variables	N	% (95%CI)^a^
Community Health Agent (CHA)	222	31.6 (28.3;35.1)
Oral health technician and dental assistant	14	2.0 (1.2;3.3)
Dentist	91	13.0 (10.6;15.6)
Nurse	168	23.9 (20.9;27.2)
Physiotherapist	27	3.8 (2.6;5.5)
Physician	19	2.7 (1.7;4.1)
Nutritionist	11	1.6 (0.8;2.7)
Psychologist	36	5.1 (3.7;7.0)
Nursing Technician	77	11.0 (8.8;13.5)
Others	37	5.3 (3.8;7.1)

a) 95%CI: 95% confidence interval.

Table 4 shows the prevalence of symptoms related to each item of the SRQ-20. More than half (65.8%) of the participants reported feeling nervous, tense or worried, and 45.7% reported sleep-related problems.

**Table 4:** Prevalence of symptoms by groups of symptoms (SRQ-20) in health professionals (n = 702) in the Northern health macro-region of Minas Gerais state, Brazil, 2021

Groups of symptoms	Yes % (95%CI)^a^	No % (95%CI)^a^
Somatic symptoms		
Do you get frequent headaches?	46.3 (42.7;50.0)	53.7 (49.9;57.3)
Do you have a poor appetite?	17.2 (14.5;20.1)	82.8 (79.9;85.4)
Do you get poor sleep quality?	45.7 (42.2;49.5)	53.9 (50.4;57.8)
Do you experience tremors in your hands?	15.1 (12.5;17.9)	84.9 (82.1;87.4)
Do you have poor digestion?	31.3 (28.1;34.9)	68.6 (65.0;71.9)
Do you have unpleasant sensations in your stomach?	30.1 (26.8;33.6)	69.9 (66.4;73.2)
Depressive/anxious mood		
Do you get scared easily?	45.4 (41.7;49.0)	54.6 (50.9;58.3)
Do you feel nervous, tense or worried?	65.8 (62.2;69.2)	34.2 (30.7;37.8)
Have you been feeling sad lately?	41.5 (37.8;4.1)	58.5 (54.8;62.1)
Have you been crying more than usual?	23.2 (20.1;26.4)	76.8 (73.5;79.8)
Decreased vital energy		
Do you find it difficult to think clearly?	31.1 (27.7;34.6)	68.9 (65.3;72.2)
Do you find it difficult to perform your daily activities with satisfaction?	35.0 (31.8;38.9)	64.7 (61.0;68.1)
Do you find it difficult to make decisions?	36.4 (32.9;40.0)	63.6 (60.0;67.1)
Do you have difficulties at work (is your work hard, does it make you suffer?)	18.4 (15.6;21.5)	81.6 (78.5;84.3)
Do you feel tired all the time?	37.7 (34.1;41.3)	62.3 (58.7;65.9)
Do you get tired easily?	45.4 (41.7;49.0)	54.6 (50.9;58.3)
Depressive Thoughts		
Do you feel unable to play a useful role in your life?	15.1 (12.6;17.7)	84.9 (82.0;87.3)
Have you lost interest in things?	28.7 (25.4;32.1)	71.3 (67.9;74.6)
Do you feel useless, worthless?	9.4 (7.4;11.8)	90.6 (88.2;92.5)
Do you have thoughts about ending your life?	3.6 (2.5;5.3)	96.4 (94.6;97.5)

a) 95%CI: 95% confidence interval.

In the final model of multiple analysis, the following variables remained with statistical significance (p-value ≤ 0.05): previous (PR = 2.42; 95%CI 1.43;4.08) and current (PR = 1.54; 95%CI 1.25;1.89) mental disorders symptoms; overwork during the pandemic (PR = 1.42;95% CI 1.16;1.73); previous symptoms of anxiety (PR = 1.27; 95%CI 1.01;1.61); depression (PR = 1.27; 95%CI 1.06;1.52); and other previous symptoms of mental disorders (PR = 1.20; 95%CI 1.01;1.43) (Table 5). VIF values below 10 and tolerances above 0.20 for each variable indicated the absence of multicollinearity.

**Table 5:** Results of the multiple Poisson regression with robust variance between common mental disorders and study variables in health professionals (n = 702) from the Northern health macro-region of Minas Gerais state, Brazil, 2021

Variable^a^	PR (95%CI)^b^	p-value
Have you ever had symptoms of mental disorders		
No	1.00	
Yes	2.42 (1.43;4.08)	0.001
Are you currently experiencing symptoms of mental disorders		
No	1.00	
Yes	1.54 (1.25;1.89)	< 0.001
Work during the pandemic		
I worked the same way or less than usual	1.00	
I worked more than usual and I felt overwhelmed	1.42 (1.16;1.73)	< 0.001
Previous self-reported anxiety symptoms		
No	1.00	
Yes	1.27 (1.01;1.61)	0.049
Previous self-reported depressive symptoms		
No	1.00	
Yes	1.27 (1.06;1.52)	0.007
Other previous self-reported symptoms		
No	1.00	
Yes	1.20 (1.01;1.43)	0.031

a) Variables that remained in the final model, with p-value ≤ 0.05; (b) PR (95%CI): prevalence ratio (95% confidence interval).

## DISCUSSION

CMDs were prevalent in about four out of ten health professionals in the sample consulted. In the final model, the factors associated with the presence of CMDs among the study professionals were previous and current symptoms of mental disorders, work overload during the COVID-19 pandemic, previous symptoms of anxiety, depression and other previous symptoms of mental disorders.

The overall prevalence of CMDs found among PHC professionals in Montes Claros was similar to that of a survey conducted in 2013, with CHA in the same municipality of Montes Claros;^18^ ^)^ and with a percentage higher than that found in a survey conducted in 2017, with primary health care professionals in the municipality of Diamantina, also in Minas Gerais;^19^ in addition to that of a study conducted in a 2005 with PHC workers in 41 municipalities in the South and Northeast regions of the country.^9^

No studies were identified in Brazil that evaluated the prevalence of CMDs in PHC professionals in the COVID-19 pandemic period selected for this study; however, there are similar findings related to this topic in other international studies.

A cross-sectional study from a cohort of just over 4,000 health professionals in the United Kingdom,^20^ found a 58.9% prevalence of CMDs. A study conducted in the United States found symptoms of depression and anxiety in 24.0% of the sample, and posttraumatic stress disorder in 30%.^21^ However, the instrument used to measure this variable in both studies was not the SRQ-20 but the General Health Questionnaire (GHQ-12), in addition to the fact that not all the professionals taking part in the study worked in PHC.

Although there is no study conducted in Brazil on the prevalence of CMDs among PHC professionals, two studies that evaluated symptoms of anxiety and depression in nurses, were found,^14^ ^),(^ ^22^ ^),(^ ^23^ both conducted in hospitals, with prevalence ranging from 39.5% to 48.9% for anxiety symptoms, and 22.0% to 38.0% for depressive symptoms.

The prevalence analysis by groups of symptoms of the SRQ-20 showed a higher number of participants with symptoms in the “depressive/anxious mood” category, and “feeling nervous, tense or worried” stood out with high prevalence. Other studies, using the same assessment instrument, also showed a higher frequency of symptoms in this category.^9^ ^),(^ ^24^

Among female participants, almost half of them had symptoms of CMDs, and the prevalence was higher than that found in males. A systematic review and meta-analysis, focusing on health professionals during the COVID-19 pandemic in Asia,^25^ showed that females are more likely to have symptoms of CMDs such as anxiety, depression, insomnia and posttraumatic stress. These results are in line with other studies that evaluated professionals working in PHC,^8^ ^),(^ ^9^ ^),(^ ^18^ ^),(^ ^19^ although with PRs higher than those found in the present study. One of the possible explanations for the association between the variable “sex” and the CMDs, in many studies, is related to social characteristics in which, most of the time, females are responsible of doing household chores; many times, even the female professional is the only one responsible for housework and the family, in addition to suffering from gender inequality and lack of equity between women and men in the labor market, suffering from greater work overload and therefore in a condition that make them more susceptible to the risk of developing symptoms of CMDs.^26^

In this study, professionals who worked on the front line during the pandemic showed a higher prevalence of CMDs in the bivariate analysis. Among PHC professionals working on the front line in Australia,^27^ only burnout diagnosis was associated with these individuals; and in a case-control study with health professionals from China,^28^ being on the front line corresponded to an odds ratio of 2.15 for the manifestation of mental disorder.

Participants who had COVID-19 presented a higher prevalence of CMDs. There is evidence in the scientific literature on an association between COVID-19 infection and the manifestation of CMD symptoms.^10^ ^),(^ ^28^ In a cohort study that evaluated more than 60,000 people, individuals with no previous mental illness history and diagnosed with COVID-19 had a higher incidence of psychiatric disorders within 14 to 90 days after infection.^10^ A study that analyzed seven prospectively planned cohorts across six countries (Denmark, Estonia, Iceland, Norway, Sweden, and the United Kingdom), with almost 250,000 participants, showed that individuals who had COVID-19 presented a higher prevalence of symptoms of depression (PR = 1.18 - 95%CI 1.03;1.36) and poorer sleep quality (PR = 1.13 -95%CI 1.03;1.24), and those who were bedridden for more than seven days were at higher risk of symptoms of depression (PR = 1.61 -95%CI 1.27;2.05) and anxiety (PR = 1.43 - 95%CI 1.26;1.63).^29^

Previous and current self-reported symptoms of mental disorders showed an association with the presence of CMDs, both in bivariate analysis and multiple analysis. The aforementioned Australian study,^27^ revealed that 30.4% of the health professionals investigated presented with any pre-existing psychological or psychiatric symptoms, prior to the pandemic. This is a finding whose value is below the value found in this study conducted in Montes Claros, where 69.3% had symptoms of previous mental disorders.

Regarding previous and current psychological or psychiatric follow-up, both this study and the Australian study, indicated an association between these conditions and CMDs only in the bivariate analysis. Among the participants in this study with scores indicating the presence of CMDs, 40.2% did not undergo current follow-up and 35.7% did not use psychotropic medications. These results show that more than one-third of professionals with CMDs are not undergoing any types of professional treatment, which may contribute to a worsening of symptoms.

The use of psychotropic medications was associated with the presence of CMDs in the bivariate analysis, but not in the multiple analysis, differently from another study conducted with CHA in the municipality of Montes Claros, an aforementioned study,^18^ according to which the use of tranquilizers or antidepressants in the last year was 1.45 time more likely to be associated with the presence of CMDs (p-value < 0.001).

PHC professionals who worked more than usual during the pandemic period had higher prevalence ratios, when compared to those who worked less or as usual, in bivariate analysis and multiple analysis. A study conducted in Oman, a country located on the Arabian Peninsula, at the peak of the first wave of the pandemic, showed that frontline professionals, or who did not stop working during the pandemic, were 1.5 time more likely to develop symptoms of anxiety, stress and insomnia.^30^

More than half of the participants in this study reported that, at some point in their lives, they had experienced symptoms of anxiety, followed by insomnia and depressive symptoms. Symptoms of anxiety, insomnia and depression are frequent and are included in the category of symptoms that characterize CMDs.^1^

The prevalence of CMDs among PHC health professionals in the Northern health macro-region of Minas Gerais state was higher than that of other studies conducted in PHC of other locations in Brazil before the pandemic.

This is one of the first studies conducted in Brazil aimed at investigating the prevalence of CMDs among health professionals working in PHC. It is worth mentioning the limitations regarding the development of this study: (i) the cross-sectional design, which does not offer the possibility to infer a cause-and-effect relationship between the COVID-19 pandemic and the symptoms of CMDs; (ii) online data collection and snowball sampling, two strategies that have been widely used in research, including in Brazil, in the context of the pandemic;^13^ ^)-(^ ^15^ (iii) non-probability sampling, which makes the sample non-representative of the population studied, limiting inferences regarding the set of health professionals in the Northern health macro-region of Minas Gerais state, although the sample weighting strategy was used in order to reduce this limitation; as this is an exploratory study, (iv) the analysis of associated factors, that was not based on a well-defined theoretical model, although several variables that have already been explored in the literature and with potential association with the outcome investigated were taken into consideration; and (v) the healthy worker effect, a type of bias that may underestimate the prevalence of diseases studied, according to which actively employed people would be healthier, while those with some health limitation would be away from work.

Despite the limitation (iii) of the sample, especially, hinders the generalization of the results, the findings of this study reiterate the need for efficient strategies to support the mental health of professionals working in PHC. However, the impacts caused by the pandemic are being experienced, and may affect the mental health of the population, especially health professionals. In this context, we suggest health education actions on mental health, the use of instruments for detecting and monitoring symptoms of mental disorders, psychological and psychiatric support to professionals, identification of cases of work overload, elaboration of actions aimed at mental health at the end of the pandemic, in addition to strategies for preventing and preparing these professionals to cope with possible health crisis. It is also important to evaluate the working conditions of PHC health professionals to identify and modify work-related factors that may favor the emergence of CMDs such as lack of resources, devaluation of health professionals, exhausting working hours, failures in management process and excessive demands.
